# Case of Premature Development of the Mammary Glands

**Published:** 1846-12

**Authors:** 


4. *Case of Premature Development of the Mammary Glands.*—We are indebted for the following facts to Dr. JOHN DAWSON, of Jamestown, Ohio:

To-day (July 30th) Mrs. M. presented her daughter to me for an opinion as to its condition. The child is a female, nearly three years of age. It has had good general health since its birth; is of light complexion, with fair hair and rather dark eyes; is of about the usual stature of children of its age, and is the only child the mother has ever borne. Some time since, its *mammary glands* commenced being developed, and they are now about the size of large oranges, apparently well proportioned, both of the same size, with *nipples* similar to those of young ladies at the age of puberty. Neither by inquiry of the mother, nor by my own examination, could I detect any other premature development either of mind or body.—*West. Jour. of Med. and Surg.*

